# Application of X-ray topography to USSR and Russian space materials science

**DOI:** 10.1107/S2052252516003730

**Published:** 2016-03-30

**Authors:** I. L. Shul’pina, I. A. Prokhorov, Yu. A. Serebryakov, I. Zh. Bezbakh

**Affiliations:** a Ioffe Physical-Technical Institute of the Russian Academy of Sciences, St Petersburg, Russian Federation; b Research Center for Space Materials Science, Branch of the Shubnikov Institute of Crystallography of the Russian Academy of Sciences, Kaluga, Russian Federation

**Keywords:** X-ray topography, space materials science, microgravity, semiconductor single crystals, growth striations, defects, physical modelling, thermogravitational convection

## Abstract

The authors’ experience of the application of X-ray diffraction imaging in carrying out space technological experiments on semiconductor crystal growth for the former USSR and for Russia is reported, from the Apollo–Soyuz programme (1975) up to the present day. X-ray topography was applied to examine defects in crystals in order to obtain information on the crystallization conditions and also on their changes under the influence of factors of orbital flight in space vehicles. The data obtained have promoted a deeper understanding of the conditions and mechanisms of crystallization under both microgravity and terrestrial conditions, and have enabled the elaboration of terrestrial methods of highly perfect crystal growth. The use of X-ray topography in space materials science has enriched its methods in the field of digital image processing of growth striations and expanded its possibilities in investigating the inhomogeneity of crystals.

## Abstract 2

The application of X-ray diffraction topography in space materials science has promoted the acquisition of fundamental knowledge of space as a new technological environment. It has allowed a better understanding of the processes occurring during melt crystallization and the improvement of terrestrial methods of crystal growth for growing more homogeneous crystals.

## 1. Introduction   

X-ray diffraction imaging (XRDI) is now the effective conventional method for investigating the real structure of crystals. In modern understanding, it is associated with Lang’s work (Lang, 1957 ▸, 1959 ▸), which was published in response to the necessity of investigating the real structures of semiconductor crystals, the growth of which began at that time as an industrial method. Since then XRDI has seen intensive development (Tanner, 1976 ▸; Bowen & Tanner, 1998 ▸; Shul’pina, 2001 ▸) and it is used in many areas of science and technology. Since the 1970s, it has been actively applied in space materials science (Yue & Voltmer, 1975 ▸; Zemskov *et al.*, 1977 ▸; Ivanov *et al.*, 1979 ▸).

The importance of the problem of semiconductor crystal growth with high macro- and microhomogeneity of structure and properties stimulated intensive investigations into the processes responsible for the formation of concentration heterogeneities within them (Wang *et al.*, 2004 ▸). These investigations became an important part of technological experiments in space on semiconductor crystal growth. Using manned and automatic space vehicles (ASVs), a new technological environment defined mainly by microgravity was realised. Many processes occurring in substances in a liquid or gaseous state under these conditions are realised in different ways than on Earth. In particular, under terrestrial conditions gravitational forces prevent materials being obtained that are homogeneous with regard to distribution of components and phases. Intensive non-stationary thermogravitational convection, which is typical under terrestrial conditions, leads to an instability of the parameters of crystal growth and limits the possibility of obtaining crystals with a high degree of homogeneity and perfection of structure (Mil’vidskii *et al.*, 1997 ▸). Manned and ASVs allow us to implement the conditions of a prolonged state of weightlessness when the residual microacceleration is *g* ≃ (10^−5^–10^−6^)*g*_0_, where *g*_0_ is the terrestrial acceleration due to gravity. Under these conditions, the above-mentioned forces are considerably decreased and the corresponding limitations are removed or strongly reduced.

During space experiments on crystal growth it was supposed that the lack of thermogravitational convection would enable diffusion-controlled mass transport and consequently that more homogeneous crystals would be obtained. Experiments were mainly performed using semiconductor crystals. The electrophysical properties of semiconductor crystals are highly sensitive to the presence and character of impurities and to their structural defects. In addition, methods of investigating and controlling the real structure of semiconductor crystals had been developed, possessing high sensitivity and high spatial resolution. The concentration and structural heterogeneity of crystals reflects the heat- and mass-transfer peculiarities in the melt and is the best way to characterize both crystallization conditions and their changes. As XRDI is the most effective method of revealing and studying inhomogeneities in crystals, it started to be used actively from the very beginning of technological experiments in space.

The first space experiments (Witt *et al.*, 1975 ▸; Yue & Voltmer, 1975 ▸) already showed the fundamental possibility of growing semiconductor crystals in a microgravity environment with unique characteristics of microhomogeneity. However, judged by the set of parameters required and the reproducibility of the results, space-grown crystals at this stage could not compete with samples obtained by advanced technologies under terrestrial conditions. The use of space itself did not lead automatically to the growth of more perfect crystals. The new technological environment was more complicated than it had seemed earlier. Numerous specific factors of an ASV’s orbital flight (residual quasi-stationary microaccelerations, vibrations, the complex character of changes in small mass forces *etc.*) make an appreciable impact on crystallization processes and complicate the task of producing perfect and homogeneous crystals (Zemskov, 2001 ▸).

For a better understanding and control of the processes of heat and mass transfer in semiconductor melts, which define the uniformity of crystals grown under terrestrial and space conditions, numerical methods of calculating and modelling the hydrodynamic processes in melts (Polezhaev & Fedyushkin, 1980 ▸; Goncharov & Prokofieva, 2000 ▸; Wang *et al.*, 2004 ▸) have received intensive development. Methods of physical modelling of those perturbing factors that are characteristic of microgravity conditions and their influence on the process of crystallization have been developed (Strelov *et al.*, 2001 ▸). More sensitive methods of revealing the fine detail of dopant distribution in crystals started to become available, reflecting the specificity of heat and mass transfer in the melt. XRDI began to be applied in combination with other methods of research into crystals (selective etching, high-resolution X-ray diffractometry, electrophysical methods).

Application of the high-precision method of double-crystal X-ray topography allows one to carry out a quantitative assessment of the concentration and features of the dopant distribution in a crystal. In particular, quantitative plane-wave X-ray topography has been effectively applied to the analysis of the structural and compositional homogeneity of a space-grown GaSb(Te) crystal (Nishinaga *et al.*, 1997 ▸; Voloshin *et al.*, 1999 ▸; Voloshin, Lomov *et al.*, 2002 ▸; Voloshin, Nishinaga & Ge, 2002 ▸). Based on analysis of the XRDI data, it was established that the crystal was grown under almost fully diffusion-controlled mass transport. It was also shown that the structural perfection of that part of the crystal that had grown without contact with the ampoule walls was substantially higher than that of comparable crystals grown under terrestrial conditions.

In work by Kartavykh (2005 ▸), Kartavykh & Ginkin (2008 ▸) and Danilewsky *et al.* (2009 ▸), the effect of microgravity on the growth of bulk InP(S) single crystals from a melt on board the ASV Foton-11 was investigated. The growth of crystals on board the satellite and on Earth (a reference crystal) was carried out by the travelling heater method. Samples of the grown crystals were investigated by metallography, double-crystal X-ray diffractometry, single- and double-crystal X-ray topography, and secondary-ion mass spectrometry. It was shown that mass transfer in the melt in microgravity is similar to the diffusion mode. During XRDI investigations of InP(S) crystals, the formation of In-based associates (clusters) in the form of spherical defects 10–20 µm in diameter was revealed. Similar large microdefects were revealed during XRDI research on a GaSb(Te) crystal which was grown on board the ASV China-14 (Voloshin *et al.*, 1999 ▸).

The use of synchrotron radiation in XRDI opened up new opportunities for assessing defects in strongly absorbing crystals. In particular, during X-ray topography investigations on an InSb(Te) crystal on the synchrotron source of the Kurchatov Institute, which had been grown by the Bridgman method on board the ASV Foton-MN2 without contact with the ampoule walls and under the periodic influence of a rotating magnetic field, the formation of growth striations, dislocations and microtwin boundaries was revealed (Senchenkov *et al.*, 2006 ▸). It was supposed that the growth striations had formed due to Marangoni convection, which inevitably arises during crystal growth without contact with crucible walls. XRDI with synchrotron radiation was used in investigations of GaSb and GaSb(In) crystals during the French experiment Eureca-AMF-118 (Duffar *et al.*, 1998 ▸). Significant lowering of the dislocation density was registered in the area of contact-free crystal growth. Synchrotron X-ray topography characterization of CdZnTe boules grown on US Space Shuttle mission USML-1 (Raghothamachar *et al.*, 1998 ▸) and of CdTe crystals grown in microgravity during the STS-95 mission (Fiederle *et al.*, 2004 ▸) demonstrated the existence of dewetting areas of the crystals and their improved quality compared with the Earth-grown reference sample. These results clearly verify that contact with the ampoule wall plays an important role in the incidence of crystal imperfections.

In the 1990s, interest in the growth of and research into protein crystals increased strongly. For many scientific purposes, and also for medicine and biotechnology, perfect and large crystals are necessary. Numerous experiments on ASVs showed that such crystals could be grown under microgravity conditions. Since 1996, XRDI methods have been applied to research on protein crystals (Dobrianov *et al.*, 1998 ▸). In work by Koishi *et al.* (2007 ▸), the conditions for obtaining X-ray topographs of protein crystals with good defect image contrast on the basis of the application of the known principles of formation of XRDI kinematic (or direct) images of dislocations were defined. It was shown that the most efficient method of obtaining high-quality images of defects in proteins is the monochromatic method in synchrotron radiation. The use of synchrotron radiation is preferable due to its high brightness, which is necessary to achieve transparency of the containers in which the crystals are usually placed, as well as due to the features of topographical images of defects in proteins caused by a weak scattering power and wide images. By means of synchrotron radiation, topographs of tetragonal lysozyme crystals were obtained (Voloshin *et al.*, 2012 ▸).

In this work, the authors’ experience of the use of XRDI is reported for investigations of crystals grown during space flights on Soviet and Russian space vehicles, and also their terrestrial analogues, starting with the Apollo–Soyuz programme (1975). Subsequently, the authors investigated crystals of the solid solution Ge–Si–Sb (Ivanov *et al.*, 1979 ▸; Zemskov *et al.* 1977 ▸, 1979 ▸, 1984 ▸), InSb(Te) (Shul’pina *et al.*, 1981 ▸), Ge(In) (Calzadilla *et al.*, 1991 ▸), Ge(Ga) (Prokhorov *et al.*, 2005 ▸, 2009 ▸; Prokhorov, Zakharov *et al.*, 2008 ▸), Te (Parfen’ev *et al.*, 2000 ▸, 2004 ▸), GaSb(Si) (Prokhorov, Serebryakov *et al.*, 2008 ▸; Prokhorov *et al.*, 2009 ▸; Serebryakov *et al.*, 2007 ▸) and GaSb(Te) (Serebryakov *et al.*, 2012 ▸, 2014 ▸), partially or completely recrystallized under space and terrestrial conditions by the method of directional crystallization (Bridgman). The majority of these works were performed in the former USSR and in Russia and are not familiar abroad, so the goal of this paper is to familiarize researchers with the results obtained by XRDI.

## 2. Experimental: apparatus and characterization   

**Figure 1:**
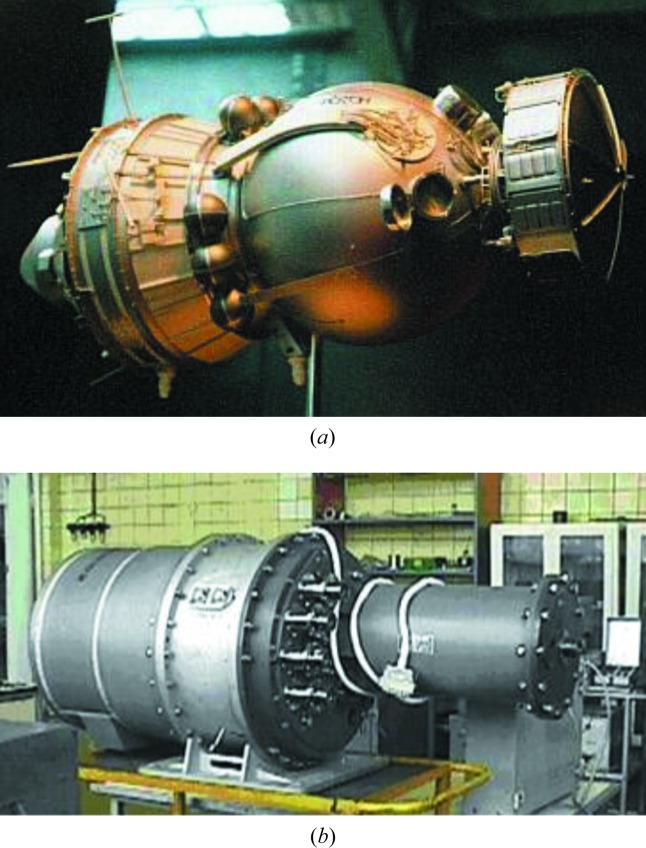
(*a*) The automatic space vehicle Foton for technological experiments in a microgravity environment. Outer diameter 2.5 m, mass 6200 kg, active lifetime 18 d, maximum distance from Earth surface (apogee) 400 km, minimum distance from Earth surface (perigee) 200 km, initial orbital period 90.5 min. The level of residual low-frequency microaccelerations (within the frequency range 0.001–0.1 Hz) is 1–7 × 10^–6^ g_0_. (*b*) The multizone electrovacuum furnace Polizon for technological experiments on crystal growth in space.

A considerable proportion of the Russian experiments under flight conditions were carried out on the ASV Foton (Fig. 1 ▸ *a*). The characteristics of this ASV are specified in the figure caption. Crystal growth under the conditions of microgravity was carried out in quartz ampoules in the multipurpose Polizon furnace (Fig. 1 ▸ *b*). Starting crystals were grown by the Czochralski method. Cylindrical samples with a diameter of 23 mm and a length of 75 mm, intended for carrying out experiments on board the ASV and for postflight research on Earth, were cut from them. An ampoule containing the sample was put into a gradient furnace with programmed heating. The furnace’s mode of operation was calculated in such a way that a part of the sample remained unmelted and later acted as a seed in the crystallization of the melted part, after the necessary soaking and controlled temperature lowering (Serebryakov *et al.*, 2007 ▸, 2012 ▸).

For the physical simulation of microgravity conditions on Earth, special installations were used. A schematic diagram of one of the last modifications is provided by Sidorov *et al.* (1999 ▸). The central part of the installation was the furnace, which was intended for crystal growth by the vertical Bridgman method with axisymmetric top heat input. Crystal growth was carried out in the axial temperature profile moving at a constant rate (with no movement of the sample or heater), which excluded uncontrolled vibrations arising from the operation of the movement mechanisms. The installation was equipped with devices permitting the imitation of various perturbation factors in the melt during the process of crystal growth, imitating flight conditions.

The specimens for these investigations were plates which were cut from near the centre along the longitudinal axis of the crystal, and they contained both seed and regrown part. The thickness of each specimen under investigation was defined according to the absorption of X-rays in the crystal and the research technique used. As all crystals studied by us possessed high absorption, it was necessary for their investigation by transmission topography methods that the thickness of each specimen after removal of the damaged layer had to be no more than 450 µm (μ*t* ≃ 10, where μ is the linear photoelectric absorption coefficient and *t* is the specimen thickness).

Such specimens could be investigated by the anomalous X-ray transmission technique in a wide incident beam of Mo *K*α radiation, or in a narrow beam of Mo *K*α radiation with scanning of the specimen and film similar to Lang’s method (Tanner, 1976 ▸; Bowen & Tanner, 1998 ▸; Shul’pina, 2001 ▸).

We also used a back-reflection method (Bragg’s) with scanning of the specimen and film in a narrow beam, and a slit in the non-transparent screen before the film to pass the reflected beam (Shul’pina, 2001 ▸). With Cu *K*α radiation, the asymmetric reflections were used as a rule, allowing us to achieve the best defect image contrast. They were characterized by a combination of a large penetration depth (7–15 µm) of X-rays (thickness of informational layer) and a small width of the rocking curve (5–15 arcsec) for the used reflection. To strengthen the defect image contrast in the symmetric reflections, Mo *K*α radiation was used.

One of the most widely used sensing techniques for investigating the concentration micro-inhomogeneity of crystals in the form of growth striations is the double-crystal method in the (**n**, −**n**) nondispersive crystal setting (Tanner, 1976 ▸; Bowen & Tanner, 1998 ▸). However, the majority of crystals exhibit worse structural perfection than crystals of germanium and silicon. In this regard, to expand the beam and form a near-plane wave, we used strongly asymmetric reflections of 511 and 620 type (the factor of asymmetry of these reflections is *b* ≃ 0.01) from monochromators made from highly perfect dislocation-free germanium. Experiments on these crystals were conducted according to the (**n**, −**m**) scheme in reflections, well consistent with the Bragg angle due to reflection from a monochromator. Filming was carried out in the working point on the rocking curve, corresponding to 50% of intensity from the peak value. For investigations of Ge crystals (Ge is widely used in space materials science as a model material), the most sensitive nondispersive scheme of diffraction in the parallel (**n**, −**n**) setting of crystals, corresponding to the quasi-plane wave XRDI method, was implemented.

Topographs were interpreted taking into account both the approximations of the dynamic and kinematic theories of diffraction images, and the experience that has been accumulated by the authors in their research on a large number of various crystals. The basic principles of these theories are considered within certain references (for example, Authier, 2001 ▸) and are not discussed here.

## 3. Main results   

### 3.1. Results of early experiments in space   

In the field of space materials science, XRDI has been used actively since the first experiments on crystal growth in space (Yue & Voltmer, 1975 ▸; Zemskov *et al.*, 1977 ▸). In particular, XRDI was used in the investigation of crystals of Ge–Si–Sb solid solutions obtained in the course of the preflight preparation, orbital flight and imitating experiments which were carried out according to the Soviet part of the experimental Apollo–Soyuz program (1975) (Zemskov *et al.*, 1977 ▸; Ivanov *et al.*, 1979 ▸). The purpose of the imitating experiments was the identification of the influence of the gravitational vector relative to the crystallization direction on the distribution of components of a solid solution in the course of growth under terrestrial conditions. The structures of longitudinal sections of crystals of the same composition (Ge + 1 at.% of Si and + 0.001 at.% of Sb) were studied. The crystals were grown by three variations of the Bridgman method: with the crystal oriented horizontally, so the force of gravity is directed perpendicular to the direction of crystallization, and also with the ingot oriented upright and the hot zone either below or above, so the force of gravity is directed parallel or antiparallel to the direction of crystallization. Comparison of the topographs obtained by the reflection method in Cu *K*α radiation showed that the crystals grown by the horizontal crystallization technique were the most imperfect in their structure, while the most perfect crystals were grown by vertical crystallization with the hot zone on top. This was manifest in the various widths of the initial and most imperfect crystallization area, and in the intensity of manifestation of growth striations (Ivanov *et al.*, 1979 ▸). Subsequently, the variant of vertical crystallization with the hot zone on top was chosen as the basis of the technology for growing the most homogeneous crystals in terrestrial conditions.

In space-grown crystals the growth striations were not observed, although in general their structure was much more imperfect than in the Earth-grown analogues. In the cross-sections of the flight crystals, strongly asymmetric macroinhomogeneity relative to the longitudinal axis was revealed (Ivanov *et al.*, 1979 ▸; Zemskov *et al.*, 1977 ▸, 1979 ▸), with segregation of Si and Sb in diametrically opposite directions. Compared with the results of the American Skylab experiments – the growth of Ge and InSb crystals with more perfect structures than on Earth – this was unexpected and unclear. This feature was also observed in subsequent experiments on crystal growth by the floating-zone method (Zemskov, 2001 ▸), but to a lesser extent. Let us note that the structures of needle crystals of Ge with additives of Si which were grown under conditions of microgravity from a gas phase also appeared more imperfect than their terrestrial analogues, as revealed by research into their structures by XRDI methods (Zemskov *et al.*, 1984 ▸). Ge–Si–Sb solid solutions appeared to be the most sensitive to crystallization conditions. The experiments carried out on them attracted attention and stimulated intensive development of numerical computational methods and modelling of hydrodynamic processes in melts.

The physical reason for radial macro-inhomogeneity in crystals grown by the Bridgman method under flight conditions was subsequently established as a result of research into convective heat and mass transfer in melts by numerical modelling. It was established that this inhomogeneity is caused by hydrodynamic effects in melts and is connected to the action of small forces of a gravitational and inertial nature (Polezhaev & Fedyushkin, 1980 ▸; Goncharov & Prokofieva, 2000 ▸).

A number of interesting structural features of InSb(Te) crystals with grown *p*–*n* junctions, obtained by the Bridgman method under conditions of orbital flight in the Soyuz–Salyut-6 space complex, were observed and investigated by the XRDI method (Shul’pina *et al.*, 1981 ▸). In the grown crystals a so-called ‘neck’ was found, an area of crystallization with no contact with the walls of the ampoule. The structure of the crystal in this neck differed in that it showed the greatest perfection: growth striations were not observed and it had the lowest dislocation density. In InSb(Te) crystals, XRDI methods determined specific defects, namely rotation twins. Subsequently, such defects in the form of individual layers or their parallel packings were observed in other crystals grown under microgravity conditions (Nishinaga *et al.*, 1997 ▸; Senchenkov *et al.*, 2006 ▸; Voloshin *et al.*, 1999 ▸; Voloshin, Lomov *et al.*, 2002 ▸).

In a number of experiments on the crystals grown on board ASVs, gas pores were observed by XRDI methods, sometimes reaching millimetres in size (Calzadilla *et al.*, 1991 ▸). It was shown that, during complete recrystallization of tellurium under microgravity conditions, as a result of the effect of separation of the melt from the ampoule walls it is possible to obtain tellurium as a polycrystal with small grains in which abnormal positive magnetoresistance for small magnetic fields takes place. This phenomenon is not observed in bulk single-crystal and large-block tellurium specimens obtained under terrestrial conditions (Parfen’ev *et al.*, 2000 ▸, 2004 ▸).

In general, by the end of the 1990s more than 30 years’ experience of carrying out experiments on crystal growth in microgravity throughout the world showed the potential possibility of space growth of crystals with unique properties. But, at the same time, it became clear that numerous factors of orbital flight (residual microaccelerations, vibrations, the composite nature of changes of small mass forces during movement *etc.*) have a noticeable impact on the course of the crystallization process, considerably complicating the possibility of growing homogeneous and structure-perfect crystals. By this time it was understood that, under terrestrial conditions, the influence of sources of non-stationary processes is suppressed against the considerable level of thermogravitational convection. In space with a practical lack of thermogravitational convection, the main sources of disturbance are thermocapillary convection (Marangoni) and variable quasistatic and vibrational microaccelerations, the level of which is several orders of magnitude less than the acceleration due to terrestrial gravity. Detailed research into the mechanisms of influence of these disturbances became necessary. As carrying out such investigations on board spacecraft is very expensive, in the 1990s in Russia the idea arose of physical modelling of microgravity conditions on Earth (Zakharov *et al.*, 1997 ▸, 2001 ▸).

### 3.2. Characterization of crystals grown during physical modelling of the disturbing microgravity factors under terrestrial conditions   

It was shown that application of the vertical Bridgman method of crystal growth on Earth with axisymmetric heating from above lowers the level of thermogravitational convection by two or three orders, mimicking in this way the convective processes intrinsic to microgravity conditions. This idea was implemented for crystal growth of Ge doped by Ga on the automated growth apparatus Zona-03 in the mode of controlled vibration, with the possibility of measuring the impact of vibration on a melt over a wide range of amplitudes and frequencies (Strelov *et al.*, 2001 ▸). In this apparatus an axial heat supply from above was provided, with monitoring of axial and radial temperature gradients. Accelerometers recorded vibration fluctuations in three directions, axial, torsion and sloping. Specially designed hinged devices allowed the orientation of the apparatus to be changed and accordingly the axis of crystal growth relative to the direction of the gravitational force.

XRDI and Bond’s X-ray method (Bond, 1960 ▸) for the precise determination of lattice parameters, *i.e.* the measurement of reflection curves on double- and triple-crystal diffractometers in two geometries of diffraction, were used to investigate specimens in the form of longitudinal plates of (110) orientation cut from ingots along the 〈111〉 growth direction and containing a recrystallization border. X-ray diffraction data were compared with selective etching images and spreading resistance measurements (Prokhorov *et al.*, 2005 ▸).

**Figure 2:**
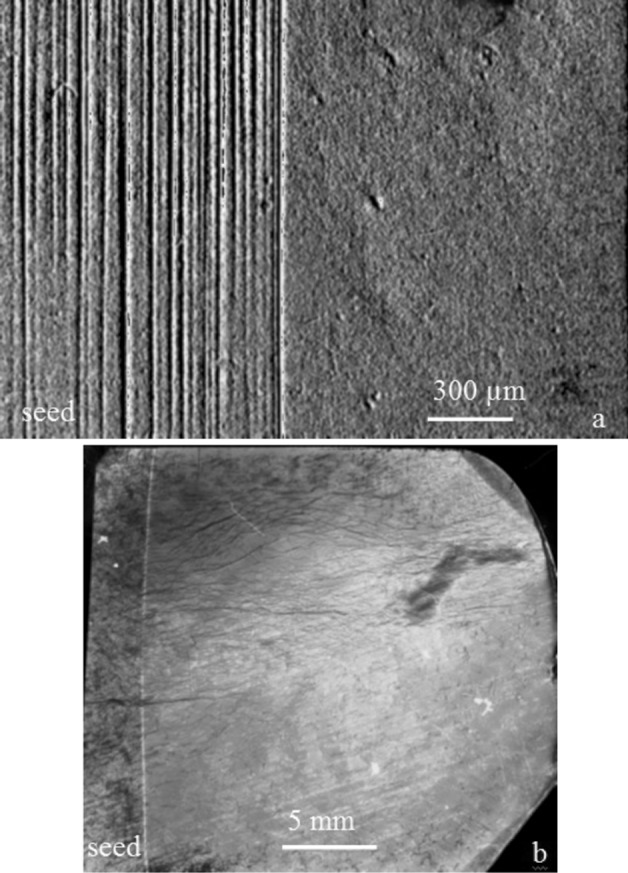
Structural features of the reference Ge(Ga) crystal. (*a*) A micrograph of the (110) longitudinal section of the crystal; stationary convection, absence of growth striations in the regrown crystal. (*b*) An anomalous transmission X-ray topograph, 220 reflection, Cu *K*α_1_ radiation.

The following main results were obtained. First of all, the high efficiency of the physical modelling of microgravity under terrestrial conditions was confirmed. During growth under conditions of weakened thermogravitational convection, growth striations in the grown crystals were not observed by either topographical methods or methods of selective chemical etching (Fig. 2 ▸). Inhomogeneity of the crystals was defined only by the presence of dislocations revealed by XRDI, but the dislocation density in the recrystallized area of the crystal was much lower than in the seed. Thus, in the recrystallized area a highly homogeneous material was created, on which it is easier to observe the individual defects due to various disturbances (Prokhorov *et al.*, 2005 ▸).

**Figure 3:**
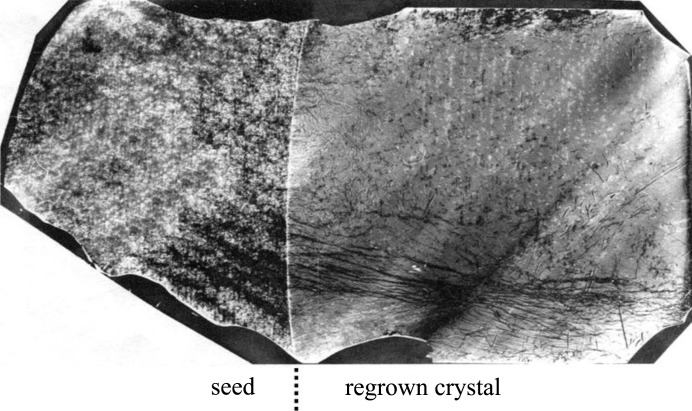
The peculiar features of a Ge(Ga) crystal with small angular boundaries, grown under torsional vibration perturbations. Anomalous transmission X-ray topograph, 220 reflection, Mo *K*_α_ radiation. The diameter of the crystal is 23 mm. The wide inclined stripes on the topograph are an artifact due to imperfection of the scanning mechanism.

It was established that the dynamic disturbances intrinsic to orbital flight have an essential impact on the microsegregation of dopant in crystals. When the torsion vibrations of a particular frequency range were applied to a melt, the formation of small-scale growth striations in crystals was observed, revealed by the method of selective chemical etching. These processes were followed by the formation of specific features of the dislocation structure of crystals connected with the formation of small-angle boundaries, slip bands and other inhomogeneities in the distribution of dislocations. Thus, a non-uniform and asymmetric distribution of individual dislocations relative to the central longitudinal axis of the crystals was observed. Small-angle boundaries made a significant contribution to the inhomogeneity of the crystals (Fig. 3 ▸). The formation of the dislocation structure was undoubtedly affected by the growth rate. The most perfect crystals were grown at a growth rate of 3.3 mm h^−1^, the least perfect at a rate of 33 mm h^−1^.

The complex character of the variation in the residual microacceleration vector relative to the crystallization front during crystal growth on board the ASV Foton is one of the major factors determining the irregularity and features of the real crystal structure (Zemskov *et al.*, 1997 ▸, 2007 ▸). In the work of Prokhorov *et al.* (2009 ▸), experiments were carried out on Ge(Ga) crystal growth under terrestrial conditions with a variation in the orientation of the growth axis relative to the gravitational force vector during crystallization. Crystal growth was started at the installation position inclined by 5° to the vertical. An hour after cooling began, the installation was returned to the normal vertical position with an angular velocity of ∼10 arcminutes s^−1^. To characterize the structural response of a crystal to disturbances introduced, the plane-wave X-ray topography technique was used, which possesses an extremely high sensitivity to small (∼10^−7^) crystal lattice deformations and, correspondingly, to very small variations in crystal composition.

**Figure 4:**
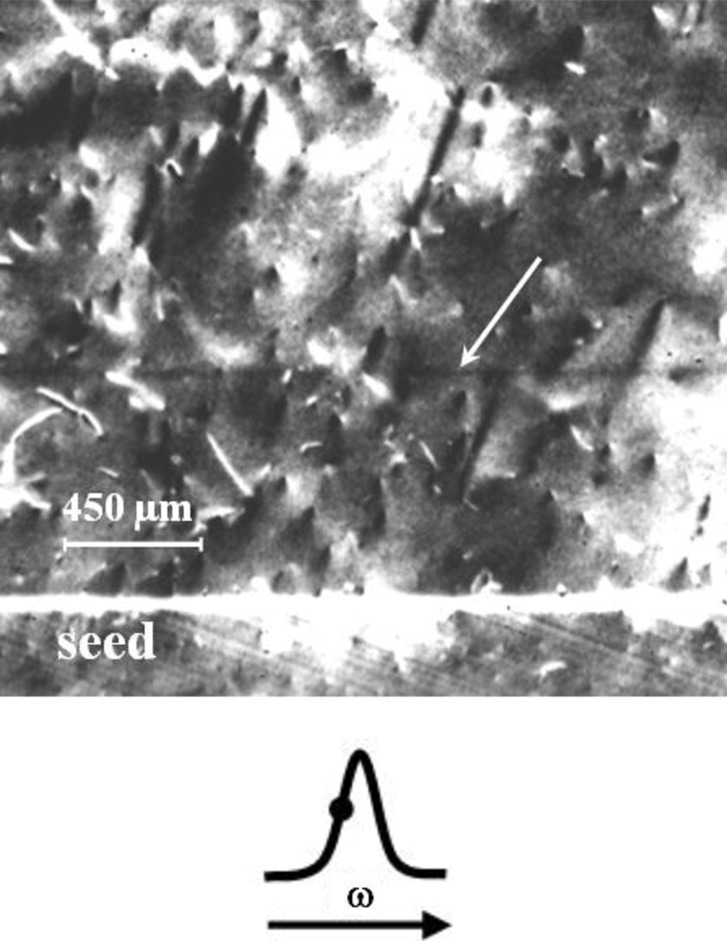
(Top) Local disruption of the dopant distribution in a Ge(Ga) single crystal under variation of the growth-axis orientation relative to the force of gravity. The rotational velocity is ∼10 arcminutes s^−1^. The structural response of the crystal to the melt disturbance caused by varying the setup orientation is revealed on the topograph by a black growth striatum (marked by an arrow). The image is a double-crystal plane wave X-ray topograph (Cu *K*α_1_ radiation, 511 reflection, ω_B_ diffraction geometry). (Bottom) The angular position of the sample during exposure (working point) is indicated by a dot.

In addition to the image of dislocations and the primary crystallization front, dopant growth striations in the seed are observed in the topograph obtained in the nondispersive (**n**,−**n**) position of crystals (Fig. 4 ▸). The image contrast for the primary crystallization front under the used conditions of measurement (with the working point located on a small-angle slope of the rocking curve and the diffraction vector **g** directed to the recrystallized part of the crystal) corresponds to the greater value of the lattice spacing in the seed, which agrees with the covalent radii of Ge and Ga atoms (*r*_Ge_ = 1.22 Å, *r*_Ga_ = 1.26 Å; Madelung, 1964 ▸) and the value for the equilibrium distribution coefficient of gallium in germanium, *k*_0_ = 0.087 (Ostrogorsky & Dragojlovic, 1995 ▸).

The structural response of a crystal to a change in orientation of the growth axis relative to the gravitational force vector during crystallization is observed in the topograph as a black stripe marked by an arrow in Fig. 4 ▸. This testifies to the change in lattice spacing of the corresponding region of the crystal caused by the local change in dopant concentration. Image contrast analysis allows the determination of the following characteristics of the region with the changed dopant concentration: the relative change (increase) in the lattice spacing is Δ*a*/*a* = 6.1 × 10^−7^ and the change (increase) in the dopant concentration is Δ*C* = 8 × 10^17^ cm^−3^. According to the results of Hall measurements, the average dopant concentration in this region for a crystal is *C* ≃ 10^18^ cm^−3^. Thus, the dopant concentration in the local region has increased by approximately twice as a result of the disturbance of the crystallization process by the variation in the orientation of the crystallization front relative to the gravitational force vector (Prokhorov *et al.*, 2009 ▸).

Thus, plane-wave X-ray topography makes it possible to obtain quantitative information about microsegregation in crystals, which is especially important in the analysis of complex crystallization processes under microgravity conditions. Let us note that the usual single-crystal XRDI methods do not allow growth striations in Ge(Ga) crystals to be revealed because of the very small lattice deformation caused by the near-equality of the Ge and Ga covalent radii (Madelung, 1964 ▸).

The conducted research showed that, under conditions of weakened thermogravitational convection, a complete similarity is not provided to microgravity conditions, but a minimization in the intensity of convective flows in a melt is possible.

Further, these results were confirmed for other semiconductor crystals of GaSb(Si) (Prokhorov, Serebryakov *et al.*, 2008 ▸; Prokhorov *et al.*, 2009 ▸; Serebryakov *et al.*, 2007 ▸) and GaSb(Te) (Serebryakov *et al.*, 2012 ▸, 2014 ▸) and used for elaborating terrestrial technologies for more perfect crystal growth.

### 3.3. Further experiments and investigations   

The high performance of XRDI was shown during investigations of a Ge(Ga) crystal grown by the floating-zone method in a microgravity environment during the ASV Foton-9 mission in 1994 (Prokhorov, Zakharov *et al.*, 2008 ▸). Because of the development of non-stationary Marangoni convection in the melt, which had a free surface, and manifestations of the facet effect (because of the large curvature of the crystallization front), the distribution of dopant in the grown crystal appeared to be extremely non-uniform. This emphasizes again that the most promising application of crystal growth in space is with the use of methods that exclude the emergence of free surfaces of the melt during the crystallization process. Application of the full range of XRDI methods (anomalous transmission of X-rays, angular scanning and double-crystal plane-wave X-ray topography) in combination with metallographic and electrophysical measurements allowed a detailed study of the structural features of this crystal and the complete restoration of the history of its growth (Prokhorov, Zakharov *et al.*, 2008 ▸).

During 2007 on board the ASV Foton-M3, an experiment on the recrystallization of a GaSb(Te) ingot under conditions excluding the development of capillary Marangoni convection was performed. Unfortunately, the depth of melting of the initial crystal was more than planned, leading to a failure of single-crystal growth and the formation of a large-block crystal structure. Comparative X-ray topographic and metallographic study of crystals grown under conditions of microgravity and on Earth did not reveal growth striations in them. However, the microhomogeneity estimated from the distribution of the spreading resistance *R*_s_ for the space-grown crystal was higher (micro-inhomogeneity coefficient *M* ≃ 8%; Serebryakov *et al.*, 2012 ▸) than for the terrestrial analogue (*M* ≃ 11%). Thus, it was shown that a decrease in intensity of convective flows in the melt, by means of excluding Marangoni convection, leads to the elimination of growth striations and an increase in microhomogeneity of the crystals, even in the case of formation of a large-block structure (Serebryakov *et al.*, 2012 ▸).

As a result of the conducted experiments, it was theoretically proven and, for the case of Ge(Ga) and GaSb(Te) crystals, experimentally implemented, that an approximation to conditions of pure diffusion mass transfer in a melt under terrestrial conditions allowed a considerable raising of the levels of macro- and microhomogeneity in the grown crystals (Serebryakov *et al.*, 2007 ▸; Strelov *et al.*, 2005 ▸).

On substrates from the GaSb(Te) crystals with increased homogeneity, high-efficiency thermophotovoltaic converters were created by the diffusion technique. In work by Serebryakov *et al.* (2014 ▸) it was shown that the characteristics of these devices (efficiency, short-circuit current, open-circuit voltage *etc.*) exceed the analogous characteristics of other samples.

### 3.4. Digital processing of X-ray topography images of growth striations   

Taking into account the features of strain of the layered inhomogeneous crystals (Hornstra & Bartels, 1978 ▸) in combination with digital processing of X-ray topography images gives information on both the peculiarities of the structural state of crystals and the disturbing factors of the crystallization process. As an example, we consider the results obtained during investigation of a highly doped GaSb(Si) crystal, which was grown during the preparation of an experiment for the ASV Foton-M3.

This GaSb(Si) crystal, which was grown by the Czochralski method in the 〈111〉 direction, was partially regrown by the vertical Bridgman method on the Polizon facility under terrestrial conditions with weakened thermogravitational convection. The design of the ampoule excluded the emergence of a free melt surface and the development of capillary Marangoni convection (Serebryakov *et al.*, 2007 ▸). The unmelted part of the ingot served as a seed; the silicon concentration was about 1–1.8 × 10^19^ cm^−3^. As a result, a single crystal was obtained with parts that differed significantly in their structure. In one part (the seed, grown by the Czochralski method with a dislocation density of *N*_D_ ≃ 10^2^ cm^−2^) growth striations were the dominant type of defect, and in another one (the regrown crystal with a dislocation density of up to *N*_D_ ≃ 10^4^ cm^−2^) the dislocations were dominant, with an absence of growth striations.

For this investigation the grown crystal was cut along its longitudinal and transverse plates. The real structure of the crystal was studied by metallographic, X-ray and electrophysical methods.

**Figure 5:**
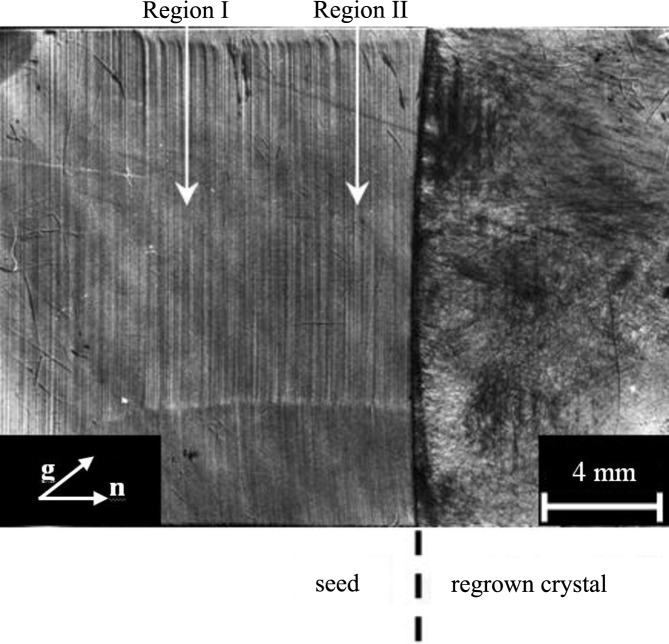
Structural features of a GaSb(Si) crystal grown by the Czochralski method (seed, on the left) and partially regrown by the vertical Bridgman method (on the right). The image is an X-ray topograph using the AXRT method, with Mo *K*α_1_ radiation and the symmetric 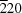 reflection. The white arrows indicate areas with different dopant states.

The main features of the seed were the presence of growth striations, macro-inhomogeneity of the dopant distribution caused by the facet effect, inclusions of the second phase and a low dislocation density (Fig. 5 ▸). The primary crystallization front had a weakly convex form towards the seed. On the seeding boundary, the formation of misfit dislocations was observed (Serebryakov *et al.*, 2007 ▸), having a linear density of 8 × 10^2^ cm^−1^, which confirmed the difference in lattice constants and, respectively, the compositions of the seed and the regrown part (RP) of the crystal. The increase in the dislocation density at the initial stage of growth up to *N*_D_ ≃ 10^4^ cm^−2^ is connected, partially, with the formation of these misfit dislocations. During the crystallization process, it has decreased in the RP to *N*_D_ ≃ 10^2^ cm^−2^.

In the RP, growth striations were absent. From the measurement results an improvement in the dopant distribution parameters was established. Both macro- and microhomogeneity of the dopant distribution in the RP were higher than in the seed and corresponded to numerical calculations (Serebryakov *et al.*, 2007 ▸).

Special attention was paid to investigating the X-ray topography images of growth striations in the seed. It appeared that it was possible to allocate regions within the seed that have essential distinctions in the images of their growth striations.

**Figure 6:**
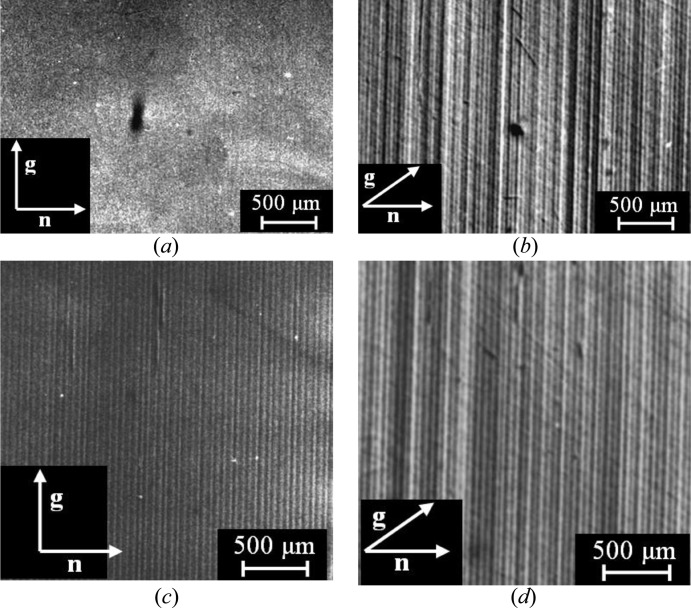
Peculiarities of the X-ray topographic images of growth striations in a GaSb(Si) single crystal. (*a*), (*b*) The strong dependence of the image contrast of growth striations in region I of the crystal (Fig. 5 ▸) on the diffraction vector **g** orientation. (*c*), (*d*) The weak dependence of the contrast of growth striation images on the diffraction vector **g** orientation in region II of the crystal. X-ray topographic images obtained using the method of anomalous transmission of X-rays, Mo *K*α_1_ radiation; (*a*), (*c*) asymmetric 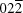 reflection, (**gn**) = 0, and (*b*), (*d*) symmetric 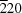, (**gn**) ≠ 0.

In region I of the crystal (Fig. 5 ▸), a strong dependence of the image contrast of the growth striations on the angle between the diffraction vector **g** and the normal **n** to the isoconcentration surface is observed (Figs. 6 ▸ *a* and 6 ▸ *b*). Such a dependence is intrinsic to a substitutional solid solution. The direction of the vector **n** is close to the crystal growth direction and determines the direction of the maximum variation in the crystal lattice spacing Δ*d*/*d* during composition variations in the growth striations.

Due to the high (at the solubility limit) dopant concentration, there is no explicit dependence of the growth striation images on the diffraction vector direction in some crystal regions (for example, region II in Fig. 5 ▸; see Figs. 6 ▸ *c* and 6 ▸ *d*). In this case, good detectability of the growth striations during exposure in the asymmetric 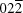 reflection (**gn** = 0) can indicate that the dopant state in these crystal regions does not correspond to an ideal substitution solid solution. In addition, the behaviour of variations in the brightness of the growth striation images along the crystal length correlates with the charge-carrier concentration distribution. In region I, where growth striations are not visible in the 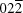 reflection, the charge-carrier concentration measured by the Hall method is approximately 20% higher than in region II with pronounced growth striations (Serebryakov *et al.*, 2007 ▸). Such a dependence can be caused by decomposition of the supersaturated solid solution of Si in GaSb during composition variations in the growth striations and the transition of the silicon fraction to an electrically inactive state. Measurements of X-ray diffuse scattering (DS) carried out using a setup with a rotating analyser at a fixed sample detuning (δθ) from the reciprocal lattice site (measurements of 2θ triple-crystal rocking curves) have revealed a slightly resolved DS peak related to low-sized crystal defects (Prokhorov, Serebryakov *et al.*, 2008 ▸). By this means, the macroheterogeneity of the seed (the presence of regions of different microstructure) in the growth direction was confirmed by X-ray, metallographic and electrophysical methods.

**Figure 7:**
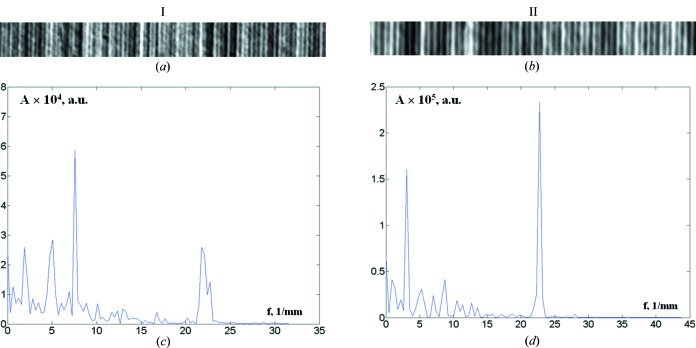
Digital processing of growth striation images in a GaSb(Si) single crystal, grown during terrestrial preparation for a space experiment aboard the Foton-M3 space vehicle. (*a*), (*b*) Fragments of topographs of region I (10 mm from the recrystallization boundary) and region II (3 mm from the recrystallization boundary), respectively. (*c*), (*d*) The corresponding spectrum densities of the intensity distribution, where *A* is the Fourier amplitude (arbitrary units) and *f* is the spatial frequency.

Fourier analysis of the useful signal of the growth striation image in a (probing) region ∼3 mm long (Fig. 7 ▸ *a*) reveals some features of the dopant distribution in the seed. In particular, the periodicity of the highest frequency signal component is close to ∼43 µm (Fig. 7 ▸ *c*), which is identical to the striation spacing on the topographs. This characterizes the well known rotation-related periodic segregation effect, common to crystals grown by conventional Czochralski pulling. In addition, the Fourier analysis detects intensity variations with periods of approximately 125, 200 and 525 µm, which are not obviously revealed in the topographic images, as well as high-frequency signal bifurcation. This suggests that the processes under study are complex and that there are several mechanisms for the formation of microscopic concentration inhomogeneities when growing crystals by the Czochralski method. It should be remarked that the low-frequency spectrum region, with a few other periodicities in addition to the dominant one, varies strongly during translation of the probing region along the crystal (Fig. 7 ▸ *d*). This characterizes slow variations in concentration and temperature fields in the growth interface region, caused mainly by turbulent convective melt flows. It should be noted that the brightness distribution of the growth striation image *I*(*x*) at a known crystal growth rate can be represented as a function of time, and frequencies can be expressed in hertz. The spacing of successive striations in this case provides information on the frequency of the perturbations responsible for their creation. This allows analysis of the response of the crystal structure to specific perturbations of crystallization during experiments, for example, aboard a spacecraft (Prokhorov, Serebryakov *et al.*, 2008 ▸; Serebryakov *et al.*, 2007 ▸). In any case, the Fourier analysis detects changes in crystal growth conditions during a certain period of time.

The results obtained give clues to knowledge of the character of the distribution and the structural state of the dopant in a crystal without the difficult simulation of image contrast. They show that the dopant distribution in a crystal is sensitive to the disturbing factors of the crystallization process, and an analysis of such distributions allows in particular the restoration of the history of crystal growth. This is especially important during the analysis of complex crystallization processes under microgravity conditions, as it allows the correlation of the observed features of the real structure of a crystal with the measured levels and frequencies of residual microaccelerations (Boguslovskii *et al.*, 2004 ▸).

**Figure 8:**
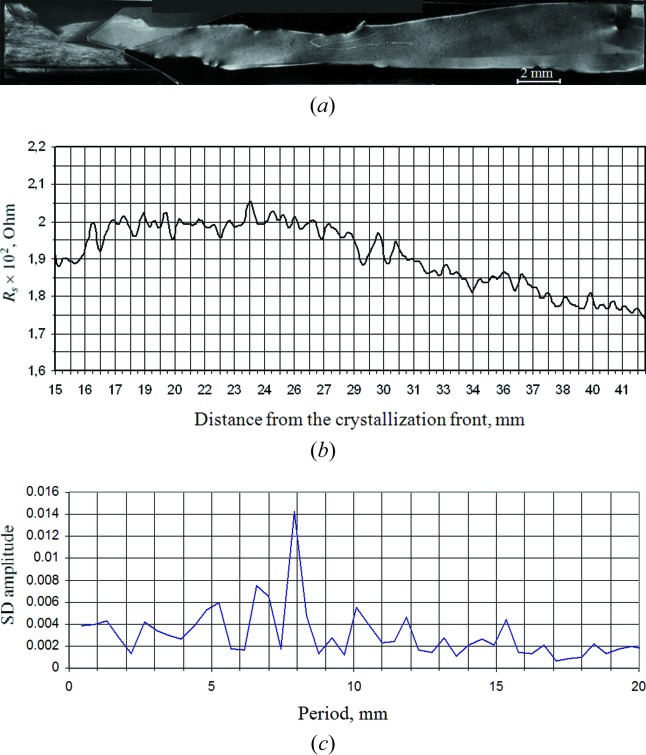
(*a*) X-ray topograph of an extended block with a length *l* ≃ 30 mm in the central part of a space-grown sample. Lang method, Mo *K*α_1_ radiation, reflection 220. (*b*) Measurement results for the spreading resistance *R*_s_ along the monoblock. (*c*) The spectral density of the *R*_s_ distribution.

In work by Serebryakov *et al.* (2012 ▸), Fourier analysis was applied to the study of the spreading resistance distribution *R*_s_ in GaSb(Te) crystals grown under space and terrestrial conditions. The *R*_s_ distribution allowed those workers to find periodicities in the dopant distribution, connected to processes of heat and mass transfer in melts in terrestrial and space environments. In the space experiment, this period in terms of time corresponded to the rotation period of the ASV Foton around Earth (Fig. 8 ▸), which confirms the influence of weak quasistationary microaccelerations on the distribution of dopant in a crystal. For the terrestrially grown crystal, the frequencies in the dopant distribution corresponded to higher-frequency processes of heat and mass transfer, intrinsic to terrestrial conditions and the weakened thermogravitational convection.

## 4. Conclusions   

The investigations carried out have shown the high efficiency of the application of XRDI methods in space materials science. On the basis of studying the real structures of crystals by XRDI methods, the features of crystals grown under the conditions of ASV flight and their differences from terrestrial analogues are explained by the difference in heat and mass transfer. From the analysis of X-ray diffraction images of growth striations, and also of the features of defect distribution in crystals, important conclusions on the influence of crystallization conditions on them, including the disturbing factors intrinsic to an ASV, can be obtained. The results obtained stimulated the development of numerical methods of calculation and modelling of the crystallization process, allowing the prediction of the results of future experiments. At present, microgravity experiments are not performed without provisional numerical calculations. The use of XRDI in space materials science has led to an improvement in XRDI methods regarding the processing of images by digital methods, which has expanded its possibilities in the investigation of inhomogeneous crystals.

The application of XRDI in space materials science has promoted the acquisition of fundamental knowledge of space as a new technological environment. It has allowed a better understanding of the processes occurring during melt crystallization and the improvement (to a certain level) of terrestrial methods of crystal growth for growing more homogeneous crystals. By the example of crystals of Ge(Ga), GaSb(Si) and GaSb(Te), it is shown that, during growth by the vertical Bridgman method under conditions of physical modelling of microgravity on Earth, it is possible to avoid the formation of segregation growth striations mainly by substantial weakening of thermogravitational convection, and thus to obtain more perfect crystals. The microhomogeneity of their dopant distribution is close to that of crystals grown under space conditions, and this allows the creation of some types of apparatus with improved characteristics on that basis.

Space experiments on crystal growth continue at the present time, and XRDI is still in intensive use in them. The main goal of these experiments is not only to grow the most perfect crystals in space, but to obtain a better understanding of crystallization mechanisms and the interactions of their multiple parameters, with a view to controlling these processes and improving them (Benz & Dold, 2002 ▸).
